# Crystal Gold for Filling Teeth

**Published:** 1854-01

**Authors:** J. Taft

**Affiliations:** Xenia, O.


*Crystal Gold for filling Teeth*, By J. Taft, Xenia, O.

Each and every profession, and pursuit in life, has its grand and fundamental points; or, that which in intrinsic value, or superior skill is requsite for its execution occupies a place *above* those things with which it is associated. Such a position does the *preservation* of the natural teeth, occupy in dental science.

The best means for the preservation of the natural teeth in a state of *perfect health*, is a matter of vital importance ; yet when decay has attacked the teeth, the question of their preservation, or restoration, if I may be allowed the term, becomes one of greater solicitude, and more urgent consideration. All are familiar with the ordinary method of arresting the decay of the teeth. Conceding the point, that almost the entire causes of decay of the teeth are external. The method of procedure is to remove those things that excite and promote decay ; remove all decomposed portions of the teeth ; give the cavity a proper form, and then fill it with the material deemed best in the case. That material almost universally acknowledged to be the best, is gold. Best, because it is less corruptable than anything else of equal adaptation, with which we are as yet acquainted. Its contrast with the teeth, in color, is but a slight objection ; it forms, when perfectly applied, a complete protection to the decayed cavity, excluding the entrance of *all* external agents, heat excepted. It is dense enough to be used even in the grinding surfaces of the molar teeth, for a great number of years. Though gold is the best material yet known for filling teeth, yet its fitness may be much modified by the mode of its preparation. The *desideratum is* the *best form* of the *best material.* Gold has hitherto been used for filling teeth, almost exclusively in the form of foil, and even the quality of this is exceedingly variable.

There are some difficulties, however, that seem necessarily connected with the use of gold in this form. The surface of the leaves are almost as smooth as if they were separately planished, and hence have no facility for welding or uniting together, so that two leaves being pressed together, only remain so because they are thus retained by the force about them. Foil that has been *perfectly* annealed after being hammered, when brought in contact under great pressure, *may* adhere *slightly*. This inability to unite, constitutes one of the strongest objections to the use of foil. The practice of introducing foil fillings in blocks, counteracts this difficulty as nearly as it is possible. Yet fillings when thus put in, will sometimes lose small portions, and when a filling *begins* to yield, it is very soon lost; "failing by piece-meal;" "coming out in fragments;" "tumbling to pieces;" &c., are remarks familiar to the ear of every dentist. A few fortunate individuals there are however, who never have such things happen in their practice, because they have a peculiar method of *their own* for performing this operation. Of course their own method is so much better than that of any one else—always succeeding most perfectlyin every operation, just because it is done in their way. It is true, that from five to twenty per cent of all the teeth filled by reputable dentists, are not successful in the first operation. And why? Sometimes because the filling was not consolidated, or because the cavity was not properly formed to retain a filling that presented a smooth surface to its hard smooth walls, and although it was consolidated perfectly as possible, gave way either in a mass, or as is frequently the case, in pieces. In regard to this point, facts will warrant the assertion, that nine-tenths of the fillings that give way, come out in pieces; not because they were not consolidated, but because they were not, and could not be united together into a solid mass. Let us imagine how greatly the probabilities of success would be increased, could we obtain some method of uniting the leaves of foil, that compose a filling into a solid mass, that could not be separated even when removed from the cavity; then success would be far more certain, notwithstanding the smooth surface of the foil is presented to the walls of the cavity. The best foil fillings that are put in by the most superior operators, when removed from the cavities, can be torn to pieces by very slight efforts, and a great many even fall to pieces before they come out of the cavities.

Cases occasionally occur, in which the teeth have such a tendency to decay, that the most perfect operations present but a feeble resistance to its progress; "never-failing dentists" to the contrary notwithstanding.

Gold in a Crystalized form has recently been introduced, and thus far seems to warrant the conclusion, that in many respects it is superior to foil. This preparation is pure, annealed, Gold Crystals. The method of its preparation precludes the possibility of its containing any alloy. The following brief remarks will be devoted to an examination of this preparation, being a mass of crystals. Under the hammer, or in the rolling mill, its particles unite together and it becomes a solid plate. When two or more pieces are pressed together, they unite so that they cannot be separated. The crystals of one part pass between, interlace, and unite perfectly, with those of another; and successive pieces may thus be made to unite to the mass till it is of any desired size.

The fitness of this preparation of gold, for filling teeth, is quite apparent when we consider all its properties. It is of such a consistence, that it can, by compression be perfectly adapted to a cavity of any form. The part of the filling presented to the walls of the cavity, is not, as with foil, a smooth, but crystalized surface; the points and edges of the crystals are presented to the walls, and take hold upon them.

Much depends upon the manner in which it is introduced, consolidated, and finished. It will at once be apparent, that the manner of filling with this preparation is different from that of foil. As the pieces that compose a filling, unite perfectly, it is not important at what point in the cavity we commence the filling; though, in this, the operator should be governed by circumstances. In crown cavities of the molar teeth, the filling can be best introduced, by beginning at the bottom of the cavity. In cavities of the approximal surfaces of the molars and bicupides, it can be introduced with greater facility by beginning the filling at that part of the cavity nearest to the neck of the tooth. In the approximal cavities of the incisors, circumstances must control the *method* of tbe operation. Instruments for filling teeth, with this preparation, must necessarily differ in some respects from those used for filling with foil. The instruments we will now describe, in connection with the manner of introducing the filling. But little need be said in regard to the preparation of the cavity; a remark or two may not be out of place here: The cavity should be *perfectly excavated* and *freed* from all decayed portions of the tooth, and extraneous matter, regardless of the kind of filling to be employed. It is not necessary to cut away the *solid* bone of the tooth, in the use of *this filling*, that we find requisite under other circumstances.

In filling, particularly approximal cavities, with foil, it is requisite to make an undercutting, that the filling may be bound together, and held in its place. This particular part of the operation, which, is attended with more or less labor to the operator; taxing the endurance of the patient, and greatly at the expense of the tooth, is not requsite, at least to the same extent, in the use of the crystal gold. In cases when there are large approximal cavities in the incisors, they are frequently ruined by this undercutting or forming the cavity properly to retain a foil filling. Now that this particular form of cavity is not requisite, in the use of the filling under consideration, will be apparent from two or three observations. The pieces of which the filling is composed, will unite together and form a solid mass, independent of the cavity by which it is surrounded. Again, this is not necessary to retain the filling in the cavity, for it has already been remarked that the filling does not present to the walls of the cavity, a smooth surface, but the ends and edges of the crystals, thereby taking hold upon the walls, and thus it is retained in place. Again, the filling in consequence of this tendency to unite to, or take hold upon the sides of the cavity, is not liable to become displaced during the operation; a vexing accident, which all operators experience, more or less, in using foil. Very large cavities cannot be filled with foil, we are told, at least very many of them; hence an excuse for the use of cement. Such cavities can be filled with crystal gold, with as much facility as smaller ones, except that a little more time is required. I do not maintain the opinion, that teeth having large cavities, would be as certainly saved, ultimately, as those having less cavities; for, it may be considered an axiom in dental science, that, other things being equal, the larger a cavity is, the less certainty there is of saving the tooth permanently. In filling lateral cavities, with *any material*, it is important that the teeth be well separated, and it is fully as necessary, and probably more so, that they be freely separated for filling with gold thus prepared. The cavity being excavated and perfectly cleansed, the gold should be cut into blocks just large enough to enter the cavity *freely*; they should not be of such a size that they require forcing into the cavity. Then with plugging forceps or pliers, place the pieces in the cavity. Two or three different forms of forceps will be sufficient for almost all conceivable cases: one pair with fine points and but slightly curved; and two pairs bent at an angle of forty-five degrees; the one with a fine point, and the other large, so that when closed, it will form a point about the size of a full medium plugger. With the slightly curved, fine pointed forcep, the blocks can be introduced into any cavities of the anterior teeth; and with those more curved, into cavities of the molar teeth; particularly crown and posterior approximal cavities. The anterior approximal cavities of the molars and bicuspides, can be ap-proached with more facility, with the forceps first described.

The forceps are only used to introduce the pieces of which the filling is composed; the compression is accomplished by the use of other instruments. The first block being introduced, a large pointed plugger is then used for setting the piece in place. This setting instrument, if I may call it such, is a large square-pointed instrument, serrated or cut upon the end; in regard to its size, we should be governed by the size of the cavity we are about to fill. It should be as large as could *freely* enter the cavity; hence quite a number of this class of instruments is required; the reason of this is apparent when we consider, that the blocks are put in as large as the cavity will receive. After the piece is set in place, then with a sharppointed instrument with an abrupt bevel, it is condensed, not however, making a smooth surface upon it, for a rough surface is required for the reception of the next piece introduced. The pieces are then introduced with the pliers, set, and condensed, successively until the cavity is full. If after the filling is compressed, it should not be as full at all points as desired ; more may be added, and made to adhere perfectly by roughning the surface, and applying as already directed. That part of the operation already described as setting, is an important item, by this, each piece is made to unite to its successor, and also to adapt itself perfectly to the space it is designed to occupy. If a block is carelessly thrown in and not fixed in place, and space be permitted under it, and an attempt then made to use the fine pointed instrument, we would fail to condense perfectly ; the instrument would only pierce the block, and probably break it to pieces ; whereas, if it is well set, no such accident can occur. After the cavity is full as it is desired to make it, the entire surface should be traversed by the fine pointed plugger, then filed off and burnished. A very beautiful finish can be put upon these fillings, with comparatively little labor. For the lateral cavities of the incisors, the same general character of points, will be required for the plugging instruments. The right and left instruments bearing these points, will enter into the variety. In addition to these, thin, flat, and even sharp edged, curved instruments, are frequently required. When the space between the incisors is small, and the blocks are made to go tightly between them, then the thin instrument will be required to introduce and place the piece in the cavity, then the same general principle will apply for condensing and finishing, already referred to.

In cases when it is desired to arch over an exposed nerve, it can be easily accomplished, with any common degree of care and skill.

It is preferable to use as large pieces as is admissible, in any given cavity ; for a large piece can be just as easily and perfectly condensed, as a small one ; quite the contrary is true in regard to foil. A much more solid filling is obtained with this preparation than with foil. In proof of this, we only refer to the fact, that a filling of it is about one fourth heavier than the same sized filling of foil. But then, upon this an objection is raised, "that it requires more gold to fill a tooth ;" so it does. I do not now intend to answer this objection, but only remark, that the more gold that can be put into a cavity of a given size, the better for the patient; and in nine cases out of ten, they will not refuse to pay for it.

Some three years ago, a preparation was introduced in New York, called "*sponge gold*," which failed to produce the desired result for filling teeth. It was altogether different from the preparation above referred to. The difference is simply this; that the crystal gold is composed of elongated crystals of annealed gold, which forms a tenacious mass, not disposed to break to pieces. Now the sponge gold, is not crystalized at all, but is gold in a state of minute granulations; possesses but feeble cohesive properties; hence it would crumble to pieces, and there would be much waste in using it. Again, it is impossible that it could unite perfectly, for it was not pure metalic gold, but partook of the character of an oxide of gold, and consequently it was liable to change after it was put into a tooth; hence the objection that subsequently arose, "that after a short time it became spongy in the tooth." This condition of it arose from two things; First, a change took place in the material itself; and second, the inability of the granules to unite. It is possible that a sponge gold, perfectly pure, may be used with some degree of success.

